# Analysis of Multiple Association Studies Provides Evidence of an Expression QTL Hub in Gene-Gene Interaction Network Affecting HDL Cholesterol Levels

**DOI:** 10.1371/journal.pone.0092469

**Published:** 2014-03-20

**Authors:** Li Ma, Christie Ballantyne, Ariel Brautbar, Alon Keinan

**Affiliations:** 1 Department of Biological Statistics and Computational Biology, Cornell University, Ithaca, New York, United States of America; 2 Section of Cardiovascular Research, Department of Medicine, Baylor College of Medicine, Houston, Texas, United States of America; 3 Department of Medical Genetics, Marshfield Clinic, Marshfield, Wisconsin, United States of America; University of California, Irvine, United States of America

## Abstract

Epistasis has been suggested to underlie part of the missing heritability in genome-wide association studies. In this study, we first report an analysis of gene-gene interactions affecting HDL cholesterol (HDL-C) levels in a candidate gene study of 2,091 individuals with mixed dyslipidemia from a clinical trial. Two additional studies, the Atherosclerosis Risk in Communities study (ARIC; n = 9,713) and the Multi-Ethnic Study of Atherosclerosis (MESA; n = 2,685), were considered for replication. We identified a gene-gene interaction between rs1532085 and rs12980554 (*P* = 7.1×10^−7^) in their effect on HDL-C levels, which is significant after Bonferroni correction (*P*_c_ = 0.017) for the number of SNP pairs tested. The interaction successfully replicated in the ARIC study (*P* = 7.0×10^−4^; *P*_c_ = 0.02). Rs1532085, an expression QTL (eQTL) of *LIPC*, is one of the two SNPs involved in another, well-replicated gene-gene interaction underlying HDL-C levels. To further investigate the role of this eQTL SNP in gene-gene interactions affecting HDL-C, we tested in the ARIC study for interaction between this SNP and any other SNP genome-wide. We found the eQTL to be involved in a few suggestive interactions, one of which significantly replicated in MESA. Importantly, these gene-gene interactions, involving only rs1532085, explain an additional 1.4% variation of HDL-C, on top of the 0.65% explained by rs1532085 alone. *LIPC* plays a key role in the lipid metabolism pathway and it, and rs1532085 in particular, has been associated with HDL-C and other lipid levels. Collectively, we discovered several novel gene-gene interactions, all involving an eQTL of *LIPC*, thus suggesting a hub role of *LIPC* in the gene-gene interaction network that regulates HDL-C levels, which in turn raises the hypothesis that *LIPC*'s contribution is largely via interactions with other lipid metabolism related genes.

## Introduction

Genome-wide association studies (GWAS) have enabled the identification of thousands of single-nucleotide polymorphisms (SNPs) that are associated with complex human diseases and traits [1]. However, most of these associated SNPs exhibit small effect sizes and collectively explain only a minor fraction of heritable variation [2]. Epistasis, or gene-gene interactions, plays an important role underlying the genetic basis of complex diseases and traits [3], [4], and has been suggested to underlie some of the “missing heritability” in human GWAS [2], [5]. High-density lipoprotein cholesterol (HDL-C) is an important risk factor for cardiovascular disease and is highly heritable [6]. In the last few years, there has been a surge of GWAS of lipid and lipoprotein traits, which have identified hundreds of loci associated with the levels of HDL-C [1], [7]–[11]. However, the combination of all significantly associated loci in large-scale meta-analyses still explains only 9.9∼12.1% of the total variance in HDL-C levels [10], [11], while 54∼70% is heritable [6]. Several recent studies have re-analyzed GWAS datasets and reported a few gene-gene interactions associated with human HDL-C levels [9], [12]–[15].

While epistasis or gene-gene interactions have long been studied in plants and animals [4], [16], they have proven difficult to identify in human GWAS mainly due to the limited statistical power caused by small effect size, moderate sample size, and the multiple-testing correction burden inherent in the large number of interaction tests required [5], [12], [17]–[20]. Prior biological insight has been incorporated into analysis of epistasis, aiming to increase power by focusing only on a selected subset of genes and SNPs that are likely to be enriched for putative interactions. Such biological insight can include, among others, previous GWAS results, protein-protein interactions, and pathway information [12], [14], [20]–[22]. By testing interactions between genetic loci that are marginally associated with lipid traits, we recently detected and replicated an interaction between *HMGCR* and a locus upstream of *LIPC* in their effect on HDL-C levels in several GWAS from different ethnicities [12].

In this study, we follow a similar approach by testing interactions between a focused set of SNPs in a Randomized Candidate Gene (RCG) study, in which the genes have been targeted as related to lipid and lipoprotein levels [13], [23]. We identified and replicated one significant gene-gene interaction affecting HDL-C levels, which involves the exact same SNP as the one previously reported to be involved in a different gene-gene interaction underlying HDL-C levels [12]. Interestingly, this SNP (rs1532085) is an expression QTL (eQTL) of *LIPC*, a gene that plays a key role in the related pathway. To further investigate this SNP's role in gene-gene interactions affecting HDL-C, we tested for gene-gene interactions between it and all other, genome-wide SNPs in large-scale data sets from the Atherosclerosis Risk in Communities (ARIC) study [24] and the Multi-Ethnic Study of Atherosclerosis (MESA; [25]. Our results combined point to several suggestive gene-gene interactions all involving rs1532085 as one of the two interacting SNPs, two of which significantly replicated in independent datasets. We discuss the possibility of *LIPC* as a hub in the gene-gene interaction network underlying HDL-C levels and the biological context of such an interaction networks.

## Materials and Methods

### Study Descriptions

All work done in this paper was approved by Cornell institutional review boards (IRB) committee. The three studies we analyzed were also approved by their local IRB committees.

### Randomized Candidate Gene (RCG) Study

**Figure 1:**
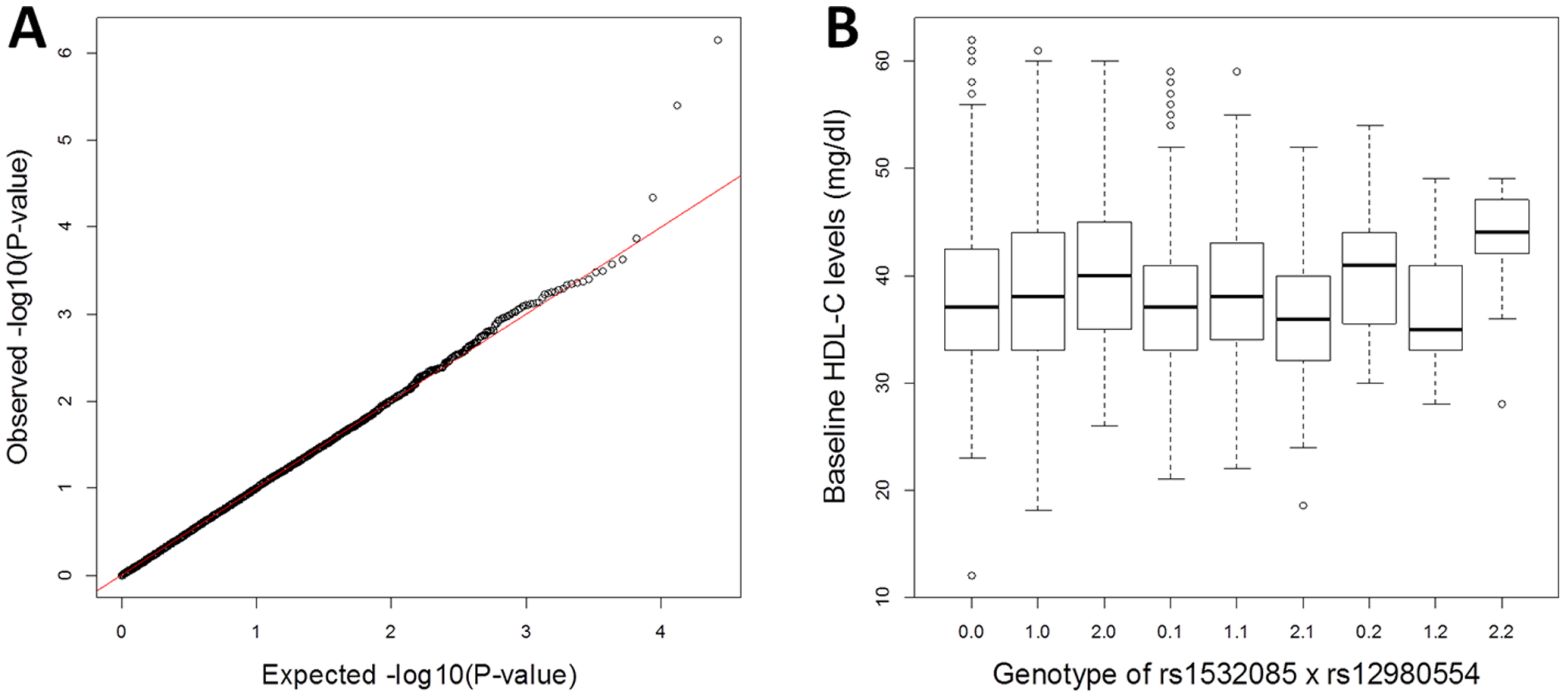
QQ plot and Box plot of the interactions identified in the RCG study. **A**) QQ plot for interaction tests between all pairs of 304 candidate SNPs in RCG. **B**) Boxplot of HDL-C levels for the nine genotype combinations of rs1532085 and rs12980554, the pair of SNPs that exhibit the most significant gene-gene interaction in this study. The interaction is dominated by the combination of AA genotype in both SNPs.

We used data from a Randomized Candidate Gene (RCG) study, which utilized a randomized clinical trial (www.clinicaltrials.gov; NCT00300482, NCT00300456, NCT00300469, NCT00300430) to assess the responses to medical treatments in individuals with mixed dyslipidemia [23], [26]. In brief, men and women with triglyceride (TG) ≥150 mg/dl, HDL-C <40 mg/dl for men or <50 mg/dl for women, and low-density lipoprotein cholesterol (LDL-C) ≥130 mg/dl were considered for the study. Baseline levels of HDL-C, TG, Apolipoprotein A1 (APOA1), Apolipoprotein B (APOB), Apolipoprotein C-3 (APOC3) and other necessary covariates were measured at the beginning of the study. A targeted set of SNPs were genotyped in or near candidate genes related to TG, HDL-C, and APOC3 pathways [27]. SNPs identified in published GWAS to be associated with HDL-C and TG [7], [28] were further genotyped. In total, 350 SNPs were genotyped in 2,091 European American (EA) individuals. We excluded samples with call rate <90%, SNPs with a call rate <90%, as well as SNPs deviating from Hardy–Weinberg equilibrium (HWE) at *P* value <0.001. After quality control (QC), 304 SNPs were kept for interaction analysis. We performed a principal component analysis using all 304 SNPs and identified no apparent population stratification [23], [26], which was also supported by observing no inflation in the QQ-plot of the *P* values for the interaction tests (Figure 1A).

### Atherosclerosis Risk in Communities (ARIC) Study

The ARIC Study is a multi-center prospective study of atherosclerotic disease [24], which recruited European Americans and African Americans from four communities: Forsyth County, North Carolina; Jackson, Mississippi; suburban areas of Minneapolis, Minnesota; and Washington County, Maryland. A total of 15,792 individuals participated in the baseline examination in 1987–1989, with three triennial follow-up examinations. We analyzed Affymetrix 6.0 SNP array genotyping of 9,713 EA from the study, and for whom data for both HDL-C levels and covariates were available. These data was first used as a replication dataset. Following our initial discovery, these data was also used for epistasis hub analysis of SNP rs1532085. For the latter analysis, after standard QC described previously (Ma et al. 2012a), we first applied linkage disequilibrium (LD) based pruning on the genome-wide autosomal SNPs using PLINK (with settings, —indep-pairwise 50 5 0.5) [29], which resulted in 136,881 SNPs that are in approximate linkage equilibrium (pairwise *r* ^2^>0.5). We then tested for pairwise gene-gene interaction in effect on HDL-C between rs1532085 and each of these 136,881 SNPs using a linear model approach described in the following.

### Multi-Ethnic Study of Atherosclerosis (MESA)

MESA is a prospective cohort study recruiting individuals aged 45–84 years from six US locations (Baltimore, MD; Chicago, IL; Forsyth County, NC; Los Angeles County, CA; northern Manhattan, NY; and St. Paul, MN) [25]. MESA aimed to determine the characteristics of subclinical cardiovascular disease and its progression. Participants were enrolled between July 2000 and August 2002 and self-reported their race group as European American, African American, Hispanic, or Chinese American. We obtained Affymetrix 6.0 SNP array genotyping of the EA samples from the database of Genotypes and Phenotypes (dbGaP; MESA SHARe, downloaded in May 2011; [30]. We considered a sample of 2,685 European Americans, for which both genotypes and HDL-C measurements were available, as a replication dataset.

### Testing of Statistical Interactions

We tested for interaction between pairs of SNPs using a previously-described linear model approach [12], [16]. Briefly, an *F*-test with four degrees of freedom was conducted to compare two models, one with marginal additive and dominance effects of the two SNPs and the other with these as well as and interactions between the two SNPs, while accounting for covariates. Hence, this test is one for pure interaction effect, on top and beyond any marginal effect of either or both SNPs. In order to increase power and false positive rate, we applied additional filtering by only considering SNP pairs for which the least frequent genotype combination, out of the 9 possible combinations, is carried by at least 10 individuals.

For each pair of SNPs, the two linear models were fitted as follows.
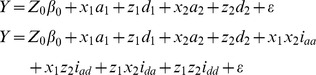
where *β*_0_ denotes a vector of intercept and covariate effects as described above, *a_i_* and *d_i_* denote the additive and dominance effects of SNP *i*, and *i_aa_*, *i_ad_*, *i_da_*, and *i_dd_* are the four interaction effects between the two SNPs, with *a* denoting additive effect and *d* dominance effect. *Y* is a quantitative trait of interest, *Z_0_* consists of the covariates, including age, sex and body mass index, *x_i_* and *z_i_* are two indicator variables of the genotype of SNP *i*, and 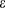 is a random error. R square value of each model was calculated and the difference of the two measures the effect of a pure gene-gene interaction, on top of any marginal effects of each SNP alone.

## Results

### An interaction between rs1532085 and rs12980554 affects HDL-C levels

We tested pairwise SNP×SNP interactions on HDL-C levels between 304 candidate SNPs in a sample of 2,091 European American (EA) individuals with mixed dyslipidemia from the RCG study. One interaction, between rs1532085 and rs12980554, was significantly associated with HDL-C levels (Figure 1A; *P* = 7.1×10^−7^; Bonferroni corrected *P*_c_ = 0.017). Importantly, rs1532085 is the exact same SNP involved in a recently-reported gene-gene interaction, though there is was in interaction with SNPs in the *HMGCR* gene [12]. While the average individual HDL-C level in the RCG study is 38.5±7.1 mg/dl, individuals that are homozygous for the minor allele of both rs1532085 and rs12980554 exhibit the highest HDL-C level of 43±5.9 mg/dl (Figure 1B).

After discovery of the gene-gene between rs1532085 and rs12980554, we further tested whether it underlies other lipid levels measured in the same study. We found it to be nominally associated with the levels of APOA1 (*P* = 2.2×10^−6^) and TG (*P* = 0.01), and almost associated with APOC3 levels (*P* = 0.08). Using a locus-based replication approach [12], we also successfully replicated this interaction on HDL-C levels in an independent European American sample from the ARIC study (*P* = 7.0×10^−4^; *P*_c_ = 0.02). Replication failed in the MESA study, which has a much smaller sample size. The replicated interaction in the ARIC study is between the same SNP upstream of *LIPC*, rs1532085, and another SNP in the second locus, rs2241589, which is proximate (22.5 kb) and in moderate LD (*r* ^2^ = 0.1) with rs12980554. This replicated interaction is also nominally affecting the level of LDL-C (*P* = 0.04) and almost that of total cholesterol (*P* = 0.07) in ARIC. Both rs2241589 and rs12980554 are located in the *KANK3*–*ANGPTL4* locus, which has been associated with HDL-C levels in European American populations [31], [32]. Interestingly, *ANGPTL4* encodes a protein that inhibits hepatic lipase's triglyceride hydrolyzing activity [33].

### Epistasis hub effect of rs1532085 on HDL-C

In a previous study [12], we reported an independent and well-replicated interaction on HDL-C levels of the same SNP (rs1532085) involved in the novel gene-gene interaction reported above. Rs1532085 has been shown to cis-regulate the expression of *LIPC* [10], [34], which encodes hepatic lipase that plays a key role in the metabolism of lipids, lipoproteins, and HDL in particular [35]. *LIPC* and rs1532085 have been associated with HDL-C levels [10]. Combining all these information, we hypothesize that *LIPC* serves as a potential hub of the epistatic interaction network regulating HDL-C levels, namely carrying out its role largely in interaction with other gene products.

**Figure 2:**
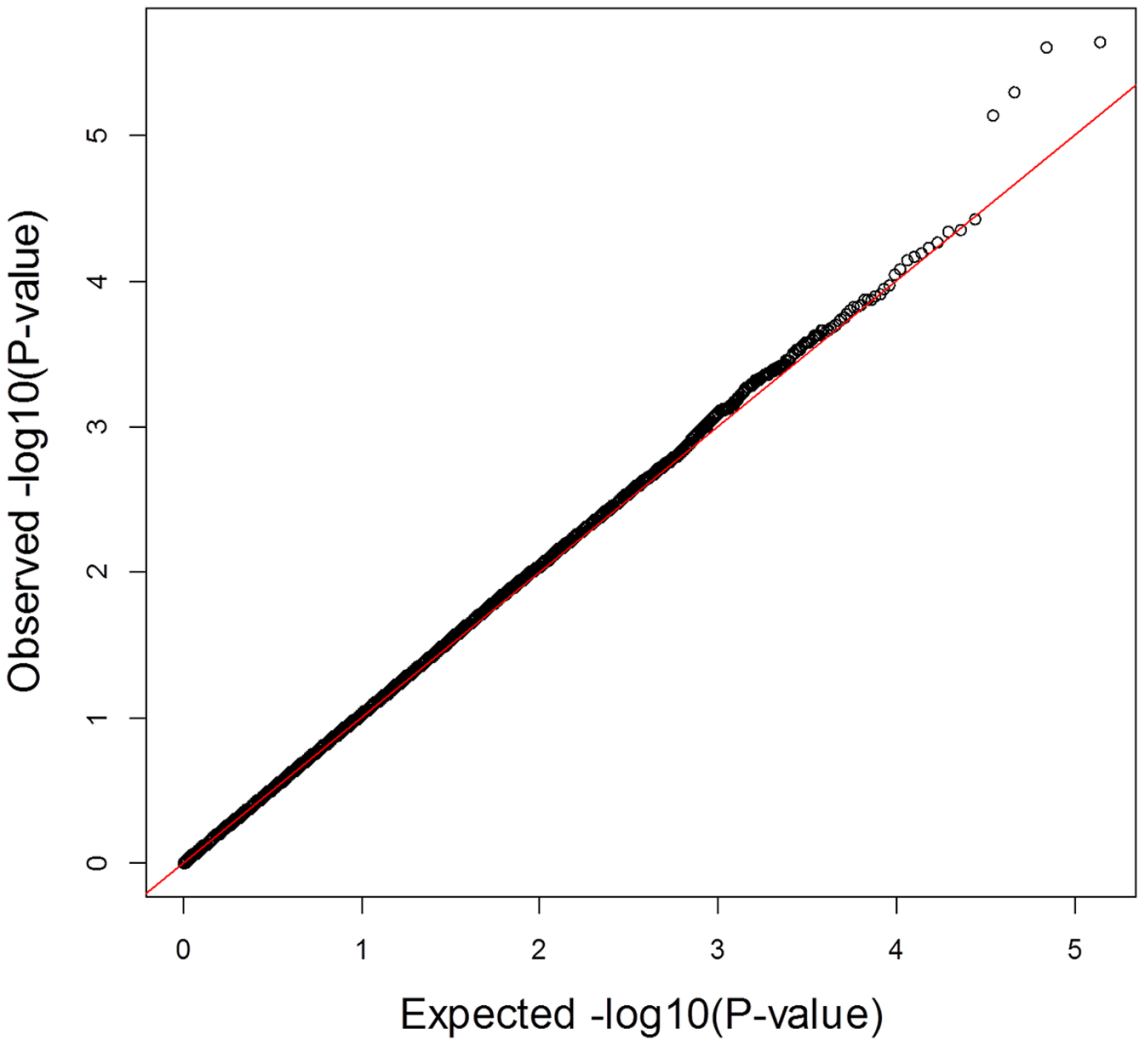
QQ plot for interaction tests between rs1532085 and genome-wide LD-pruned SNPs in the ARIC study. The plot and analysis in main text point to a deviation of the *P* values for four independent SNPs, which are detailed in Table 1.

To test this hypothesis, we tested interactions between rs1532085 and each of the other SNPs in the ARIC study in their effect on HDL-C levels. To reduce the number of dependent tests, we pruned the genome-wide autosomal SNPs based on LD [29], resulting in a subset of 136,881 independent SNPs for the interaction testing. We detected four suggestive independent interactions at a false discovery rate (FDR) of 0.25 (Table 1; Figure 2). While rs1532085 alone explains 0.65% of the total variation in HDL-C, each of these four interactions explains 0.33∼0.42% of the variation in HDL-C and combined explain an additional 1.4% variation in HDL-C for a total of 2.05% of variation in HDL-C levels. While none of the four interactions is significant by itself following a conservative Bonferroni correction, only one false positive is expected out of the four at the applied FDR of 0.25. One of the interactions, involving *HMGCR* (Table 1), has been previously reported and replicated [12]. We note that the other three interactions were not tested in that previous study since it has focused on candidate SNPs only. Hence, we attempted replicating these three interactions in an independent EA sample from MESA, and significantly replicated one of them, between rs1532085 and rs1867732 (Table 1; *P* = 0.03). The latter SNP is located in an intron of *C5orf64*, encoding an uncharacterized protein and as far as we know has not been associated with any lipid trait. Furthermore, while the interaction with another SNP, rs2901656, intronic in *C1orf105*, does not replicate, we note that *C1orf105* has been shown to be co-expressed with *C5orf64* [36], as well as with *LDLR* and *APOB* that are important players in the pathway of metabolism of lipids and lipoproteins [37]–[40].

**Table 1:** Top SNPs (FDR = 0.25) interacting with rs1532085 in affecting HDL-C levels in ARIC.

SNP	Chr	Position^a^	Gene	*P* value	R square^b^	Replication
rs2901656	1	172434812	intron of *C1orf105*	5.09×10^−6^	0.42%	N/A
rs1880966	3	74733670	163 Kb from *CNTN3*	7.29×10^−6^	0.34%	N/A
rs1867732	5	60936935	intron of *C5orf64*	2.52×10^−6^	0.36%	Replicated in MESA (*P* = 0.03)
rs2335418	5	74603479	30 Kb from *HMGCR*	2.27×10^−6^	0.33%	Reported and extensively replicated previously[12]

a dbSNP build 137.

b Difference in R square values between the model with the interaction and the model with SNP rs1532085 alone (for which R square is 0.65%), denoting the additional variance in HDL-C levels that is captured by the interaction alone.

## Discussion

In this study we have shown that rs1532085 takes part in three replicated gene-gene interactions that affect the levels of HDL-C and other lipids, with two additional interactions involving it discovered but not replicated. Beyond the statistical significance of each interaction, the fact that the same SNPs, rs1532085, has independently been discovered as part of the only significant gene-gene interaction in each of two datasets, interacting with a different SNP in each, underscores its important role in regulating HDL-C. Rs1532085 is located in the regulatory region of *LIPC* and is an eQTL of *LIPC* [34], i.e. it cis-regulates *LIPC* expression. *LIPC* is known to play an important role in the lipid and lipoprotein metabolism pathway [35]. Furthermore, *LIPC,* and rs1532085 in particular, has been associated with the level of HDL-C [8], [10], [41], total cholesterol [8], [10] and triglyceride [10], as well as with metabolic syndrome [42]. However, it explains only 0.65% of the variation in HDL-C levels, while together with its gene-gene interactions reported herein, 2.05% of that variation is explained.

One of the interactions of rs1532085, which we discovered in the RCG study and replicated in the ARIC study, is with rs2241589. The latter is intronic in *KANK3*, which is next to *ANGPTL4*. This interaction resulted in increased levels of HDL-C and APOA1, which points to increased inhibition of hepatic lipase by *ANGPTL4* when the two minor variants are present simultaneously. This interaction may also affect reverse cholesterol transport (RCT), a process in which cholesterol is removed from macrophages in the arterial wall, transferred to the HDL particle, and back to the liver for reprocessing or elimination in bile. It is one of the endogenous mechanisms to reduce atherosclerosis via the HDL particle, which plays a role in RCT, and inhibition of its function may have an effect on atherosclerosis reduction [43]. LIPC, ANGPTL4, as well as HMGCR that is involved in our previously reported interaction with rs1532085, have all been shown to physically interact or co-express with LDLR, a low density lipoprotein receptor [44].

Hubs, namely nodes in a network with a high number of connections, have been shown to be omnipresent in various types of networks, including social networks [45], protein-protein interaction networks [46], [47], and genetic interaction networks [48]. Hub genes in a genetic network tend to be important in evolution [49], and are suggested to play key roles in the biological regulation and functions [48]. Epistasis hubs have been suggested to exist in humans [50], and we propose that *LIPC* regulation constitutes such a hub in the gene-gene network affecting HDL-C levels. It is perhaps no major surprise that if a hub exists in the gene-gene interaction network underlying the levels of HDL-C, it will be related to *LIPC* and its regulation. *LIPC* encodes for the enzyme hepatic lipase which has an important role in HDL-C metabolism and RCT. Hepatic lipase hydrolyzes the HDL's phospholipids and triglycerides making it susceptible for clearance by the kidney and resulting in a net effect of HDL-C reduction.

In conclusion, while tests for detecting gene-gene interactions are not as powerful as the routine detection of marginal associations, judicious strategies of targeting SNPs that have been previously associated with lipid levels and candidate genes more generally, allowed us to identify and replicate several gene-gene interactions underlying HDL-C and other lipid levels. All these interactions involve a regulatory variant that cis-regulate *LIPC* expression, which we propose is a potential hub in the functional gene-gene interaction network underlying HDL-C metabolism and perhaps the metabolism of other lipids and lipoproteins.
